# CALCB rs3829222 T/T Genotype and Low Expression of CALCB Are High-Risk Factors for Adenoid Cystic Carcinoma of Salivary Gland

**DOI:** 10.1155/2021/5546858

**Published:** 2021-06-12

**Authors:** Chaobin Dai, Bin Zhang, Yunyang Liao, Qicai Liu, Feiguang Wu, Xiaoting Lv, Kai Zeng, Xiaofeng Zhu

**Affiliations:** ^1^School and Hospital of Stomatology, Fujian Medical University, No. 246, Yangqiao Middle Road, Gulou District, Fuzhou, Fujian Province 350000, China; ^2^Department of Oral Maxillo-Facial Surgery, The First Affiliated Hospital of Fujian Medical University, No. 20 Chazhong Road, Fuzhou, Fujian Province 350000, China; ^3^Reproductive Medicine Center, The First Affiliated Hospital of Fujian Medical University, No. 20 Chazhong Road, Fuzhou, Fujian Province 350000, China; ^4^Standardized Training Base, The First Affiliated Hospital of Fujian Medical University, No. 20 Chazhong Road, Fuzhou, Fujian Province 350000, China; ^5^Department of Respiratory, 1st Affiliated Hospital, Fujian Medical University, Fuzhou 350004, China; ^6^Department of Anesthesia, The First Affiliated Hospital of Fujian Medical University, No. 20 Chazhong Road, Fuzhou, Fujian Province 350000, China

## Abstract

**Objectives:**

To investigate the relationship between polymorphisms of calcitonin-related peptide gene II (beta-calcitonin gene-related peptide (*β*CGRP), CALCB) and serum CGRP levels in salivary adenoid cystic carcinoma.

**Materials and Methods:**

Using the polymerase chain reaction (PCR) technique, the full-length amplification and genotype analysis of CALCB genes were performed in 39 patients with adenoid cystic carcinoma of salivary gland and 158 normal controls. The gene frequencies of major genotype of CALCB in adenoid cystic carcinoma of salivary gland and normal control group were analyzed. Enzyme-linked immunosorbent assay (ELISA) was used to evaluate serum calcitonin gene-related peptide (CGRP) and its concentration of alpha and beta subtypes.

**Results:**

Univariate logistic regression analysis showed that the CALCB rs2839222 T/T genotype was closely related to the occurrence of salivary adenoid cystic carcinoma, with a correlation coefficient of 3.89.

**Conclusions:**

The serum CGRP concentration in the salivary adenoid cystic carcinoma group was 1.56 times that of the normal control group. The *α*CGRP subtype was significant, which was 3.02 times that of the normal control. The polymorphism of *β*CGRP gene is associated with genetic susceptibility to salivary adenoid cystic carcinoma, and serum CGRP and *β*CGRP can be used as novel markers of salivary adenoid cystic carcinoma.

## 1. Introduction

Salivary adenoid cystic carcinoma (SACC) is a common malignant tumor of the frontal face of the oral cavity, the pathogenesis of which is still unclear, and the biological markers closely related to the occurrence and development of SACC are currently the hot spot of clinical research. On the one hand, it can be used as a screening indicator for the early diagnosis of SACC, so as to achieve early detection, diagnosis, and treatment; on the other hand, it is important to investigate the pathogenesis and treatment options of SACC. The biological markers in serum and saliva have been validated in studies on the prognostic relevance of periodontitis and coronary heart disease [1], and some studies have found an association between serum markers and the pathogenesis of certain diseases [2, 3]. The main physiological functions of CALCB are related to the nervous system, immune system, skin system, and cardiovascular systems and are involved in a variety of physiological processes such as neurotrophy, skin immunomodulation, vasodilation, and antigen presentation. It can be used as an indicator of disease severity and therapeutic efficacy and can determine the prognosis of the disease, which has potential clinical application in treatment [4].

The biological behavior of salivary adenoid cystic carcinoma is neurological invasion, and CGRP is an important neuroimmunomodulatory peptide. Some cytokines, peptide hormones, and neurotransmitters share a two-way communication between the neuroimmune system, and CGRP is one of the mediators of the neuroimmune system's mutual regulation [5, 6]. As an endogenous immunoprotective substance, CGRP can prevent the damage caused by proinflammatory cytokines on the body after excessive activation of immune function and participate in maintaining the stable equilibrium of the internal environment of the body [7, 8]. CGRP as a local regulatory hormone is extremely low in normal human plasma but can be significantly elevated in certain lesions. Evaluation of plasma CGRP levels has become a monitoring indicator for the diagnosis or prognosis of some clinical diseases [9–13]. From this, we hypothesized that CGRP gene and homologous gene polymorphisms and serum levels are associated with salivary adenoid cystic carcinoma. The case-control study method was used to extract DNA, PCR amplification, and sequencing technology to study the *β*CGRP gene polymorphism, and the difference of serum CGRP in various patient groups and normal control group was detected by ELISA. It is expected that the relationship between CGRP gene polymorphism and its expression and SACC can be explored to provide reference significance for early clinical diagnosis.

## 2. Methods and Materials

### 2.1. Research Object

39 patients with a pathological diagnosis of adenoid cystic carcinoma of the salivary gland treated at the Department of Oral Surgery, The First Hospital of Fujian Medical University, from January 2010 to December 2013, were collected, all of whom had not received chemotherapy, radiotherapy, or other tumor-specific treatment before surgery and were excluded from distant metastases. The histopathological type and grading were based on WHO 2005 staging criteria, and the TNM staging was based on AJCC 8th edition staging criteria. Preoperative blood samples were obtained, and 10 normal parotid tissues were taken as normal controls for this experiment. All patients gave informed consent to the study and signed an informed consent form. The patients ranged in age from 29 to 65 years with an average age of 41 years. There were 27 males and 12 females.

### 2.2. Researchers

The experiments, data collection, collation, and statistics for this study were carried out by the seven authors mentioned above.

### 2.3. Determination Content of Serum or Plasma CGRP

ELISA was used to detect the levels of CGRP and its *α* and *β* subtypes in patients and normal controls (the kit was purchased from Shanghai Boyan Bioengineering Co., Ltd.).

### 2.4. The Extraction of DNA

In the case of patient's informed consent, the DNA was extracted according to the procedure of the kit, and the whole blood DNA was stored in a refrigerator at -20°C by separating the proteins, centrifuging, and filtering.

### 2.5. PCR Amplification

The primer was designed by the biosoftware Primer Premier and biosynthesized by Beauchamp; upstream primer 5′-CGC ATC TGT ACC TTG CAA CT-3′ and downstream primer 5′-TCA AAT TCC CGC TCA CTT TA-3′ add to the PCR reaction system. The agarose gel was prepared with 0.5XTBE buffer. After amplification, the target fragment PCR amplification product of each sample gene was loaded into different sample tanks of the same gel and electrophoresed. The PCR results were judged by standard molecular weight comparison, and the gel scanning system was imaged. The purified PCR product was stored in a refrigerator at -20°C for use.

### 2.6. Product Purification and Sequencing

The PCR product was sent to Hangzhou Boshang Bioengineering Co., Ltd. for purification and sequencing.

### 2.7. Statistical Analysis

The frequencies of *β*CGRP alleles and genotypes were analyzed using SPSS 20.0 for correlation between genotype and risk of disease using univariate logistic regression analysis.

## 3. Results

### 3.1. Sequencing Results of Various Genotypes of *β*-Calcitonin Gene in Salivary Adenoid Cystic Carcinoma and Its Control Group (Figure 1)

### 3.2. Genotypic Frequency Distribution of *β*CGRP Gene rs11603873 T/T and rs79501047 A/G Polymorphisms in Disease Group and Normal Control Group

The *β*CGRP gene polymorphisms (rs11603873 T/C and rs79501047 A/G) are common in the Han population, and the rs11603873 C genotype has an increased risk of salivary adenoid cystic carcinoma compared with the rs11603873 T genotype, 2.27 times, and the rs79501047 G genotype was 3.76 times that of the rs79501047 A genotype (Table 1).

### 3.3. Differences in Serum CGRP Concentration between Disease Group and Normal Control Group

**Figure 2:**
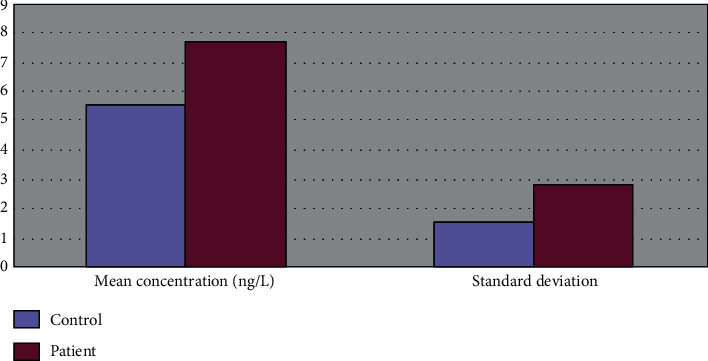
Difference map of CGRP determination of patients and their control groups.

The average serum CGRP concentration in patients with salivary adenoid cystic carcinoma was 1.40 times that of the normal control group (Figure 2).

## 4. Discussion

In 1983, Rosenfold et al. used genomic recombination and molecular biology techniques to isolate CGRP, confirming the presence of CGRP in both humans and animals [14–16]. CGRP and calcitonin (CT) are derived from the same gene and are members of the calcitonin gene-related peptide family. CGRP consists of 37 amino acids with a molecular weight of 3786.91. Its gene consists of 2800 base pairs, including 5 introns and 6 exons. It is processed by transcriptional polyadenylation and selective RNA splicing. The formation of CGRP mRNA in neural tissue is further translated into CGRP to exert biological effects. There are two isomeric peptides with similar molecular structure and biological activity in human and rat, which are, respectively, called *α*CGRP and *β*CGRP [17].

Adenoid cystic carcinoma is a common malignant tumor of the salivary glands in the head and neck. Studies have shown that neurotropic metastasis is related to the fusion of multiple genes in the host and gene mutations. There is no mention of the incidence-related genotype [18]. In lung cancer research, it was found that the CALCB gene polymorphism is associated with the incidence of lung cancer [19]. In this study, we found that the CALCB rs2839222 T/T genotype is closely related to the occurrence of salivary adenoid cystic carcinoma, which explains the genetic susceptibility of salivary adenoid cystic carcinoma to some extent.

As a local regulatory hormone, CGRP is extremely low in normal human plasma, but it can be significantly elevated in some lesions. Evaluation of plasma CGRP content has become a monitoring index for clinical diagnosis or prognosis. Takami et al. used radioimmunoassay for the first time in normal human plasma CGRP content of 6.7 ± 3.0 pg/ml (M ± SD), while with medullary thyroid carcinoma (MTC) patients with elevated plasma CGRP content, they believe that plasma CGRP is MTC. One of the body fluid markers, the determination of plasma content is important for the diagnosis of tumors, malignancy judgment, and prognosis [17]. Entschladen [20] et al. found that cancer-associated neurogenesis likely promotes the development of neural infiltration of cancer cells and that CGRP is an important neuroimmunomodulatory peptide; therefore, the link between CALCB and its encoded production of CGRP and SACC is worth exploring. Literature reports that CALCB stimulates the proliferation of T lymphocytes [21]. And the immunomodulatory effect of CALCB in the skin system, we speculate that in tumorigenesis, the tumor tissue methylates the CALCB gene through a series of pathways and reduces the expression of CALCB protein, thereby reducing the proliferation stimulation of T lymphocytes, in order to achieve escape the effect of cellular immunity [22, 23]. It has been found that a secreted peptide encoded by CALCB promotes the growth of Ewing sarcoma. Targeting the CALCBRAMP1 axis inhibits growth of Ewing sarcoma. The expression of CALCB protein is mainly distributed in the cytoplasm, suggesting that the change of CALCB protein expression level can be studied from the morphological point of view, which can distinguish the tissue cell specificity of the gene expression difference and make up for the deficiency and deficiency of other research methods [17]. Recent studies have shown that hypermethylation of the CALCB gene promoter is an important marker for the development of malignant tumors such as esophageal cancer, colon cancer, and thyroid cancer [24–26]. The main physiological functions of CALCB involve many systems, such as nervous system, immune system, skin system, and cardiovascular, participate in various physiological processes such as neurotrophy, skin immune regulation, vasodilation, and antigen presentation, and participate in the occurrence and development of diseases. It can be used as a judgment indicator of disease severity and treatment effect, can judge the prognosis of the disease, and has potential clinical application value in treatment [4]. Loss of CALCB protein expression may be an important marker for the progression of salivary adenoid cystic carcinoma.

In summary, serum and salivary markers have been shown to have a diagnostic role in a number of diseases. In the present study, a purposeful experimental design based on previous studies on CALCB and the neurotropic aggressive qualities of SACC was conducted, and it was concluded that CALCB gene polymorphisms are associated with genetic susceptibility to salivary gland adenoid cystic carcinoma and that serum CGRP and *β*CGRP can be used as novel markers for salivary gland adenoid cystic carcinoma, which provides some ideas to help in the early diagnosis and updating of treatment options for SACC in the clinic.

## Figures and Tables

**Figure 1:**
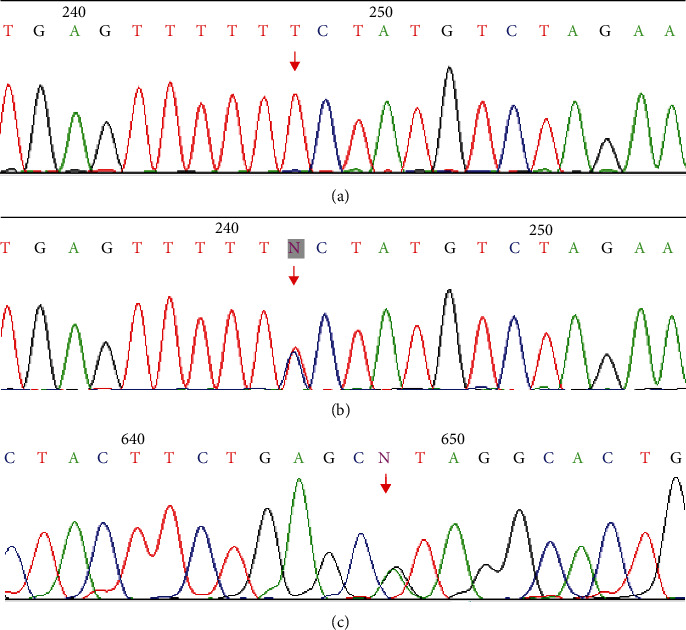
Sequencing results of some patients with adenoid cystic carcinoma of the salivary gland. (a) *β*CGRP gene rs11603873 T/T genotype. (b) *β*CGRP gene rs11603873 C/T genotype. (c) rs79501047 A/G genotype.

**Table 1:** Relationship between common polymorphisms of *β*CGRP gene and risk of salivary adenoid cystic carcinoma.

	rs11603873 (C:T)			rs79501047 (G:A)		
	T	C	OR	A	G	OR
Disease group	26	58	3.27	20	64	3.76
Control group	150	102		136	116	

## Data Availability

Data is available upon request from the authors.
